# High-Yield HIV Testing, Facilitated Linkage to Care, and Prevention for Female Youth in Kenya (GIRLS Study): Implementation Science Protocol for a Priority Population

**DOI:** 10.2196/resprot.8200

**Published:** 2017-12-13

**Authors:** Irene Inwani, Nok Chhun, Kawango Agot, Charles M Cleland, Jasmine Buttolph, Harsha Thirumurthy, Ann E Kurth

**Affiliations:** ^1^ Kenyatta National Hospital, University of Nairobi Nairobi Kenya; ^2^ Yale University School of Nursing Orange, CT United States; ^3^ Impact Research and Development Organization Kisumu Kenya; ^4^ New York University Rory Meyers College of Nursing New York, NY United States; ^5^ Peace Corps Washington, DC United States; ^6^ University of Pennsylvania Philadelphia, PA United States

**Keywords:** HIV, adolescents, female, youth, viral load, Kenya, self-testing, cash transfer, SMS message

## Abstract

**Background:**

Sub-Saharan Africa is the region with the highest HIV burden. Adolescent girls and young women (AGYW) in the age range of 15 to 24 years are twice as likely as their male peers to be infected, making females in sub-Saharan Africa the most at-risk group for HIV infection. It is therefore critical to prioritize access to HIV testing, prevention, and treatment for this vulnerable population.

**Objective:**

Using an implementation science framework, the purpose of this research protocol was to describe the approaches we propose to optimize engagement of AGYW in both the HIV prevention and care continuum and to determine the recruitment and testing strategies that identify the highest proportion of previously undiagnosed HIV infections.

**Methods:**

We will compare two seek recruitment strategies, three test strategies, and pilot adaptive linkage to care interventions (sequential multiple assignment randomized trial [SMART] design) among AGYW in the age range of 15 to 24 years in Homa Bay County, western Kenya. AGYW will be recruited in the home or community-based setting and offered three testing options: oral fluid HIV self-testing, staff-aided rapid HIV testing, or referral to a health care facility for standard HIV testing services. Newly diagnosed AGYW with HIV will be enrolled in the SMART trial pilot to determine the most effective way to support initial linkage to care after a positive diagnosis. They will be randomized to standard referral (counseling and a referral note) or standard referral plus SMS text message (short message service, SMS); those not linked to care within 2 weeks will be rerandomized to receive an additional SMS text message or a one-time financial incentive (approximately US $4). We will also evaluate a primary prevention messaging intervention to support identified high-risk HIV-negative AGYW to reduce their HIV risk and adhere to HIV retesting recommendations. We will also conduct analyses to determine the incremental cost-effectiveness of the seek, testing and linkage interventions.

**Results:**

We expect to enroll 1200 participants overall, with a random selection of 100 high-risk HIV-negative AGYW for the SMS prevention intervention (HIV-negative cohort) and approximately 108 AGYW who are living with HIV for the SMART design pilot of adaptive linkage to care interventions (HIV-positive cohort). We anticipate that the linkage to care interventions will be feasible and acceptable to implement. Lastly, the use of SMS text messages to engage participants will provide pilot data to the Kenyan government currently exploring a national platform to track and support linkage, adherence to treatment, retention, and prevention interventions for improved outcomes.

**Conclusions:**

Lessons learned will inform best approaches to identify new HIV diagnoses to increase AGYW’s uptake of HIV prevention, testing, and linkage to care services in a high HIV-burden African setting.

**Trial Registration:**

ClinicalTrials.gov NCT02735642; https://clinicaltrials.gov/ct2/show/NCT02735642 (Archived by WebCite at http://www.webcitation.org/6vgLLHLC9)

## Introduction

### HIV in Adolescent Girls and Young Women

Adolescents are at significant risk for HIV infection, and despite the need to increase knowledge of HIV status in this priority population, access to and uptake of testing remain low [1-3]. Globally, sub-Saharan Africa remains the region with the highest HIV burden, and infections disproportionately occur among adolescent girls and young women (AGYW) in the age range of 15 to 24 years [4]. Adolescent girls and young women are twice as likely as their male peers to be infected, making females in sub-Saharan Africa the most at-risk group for HIV infection [4]. High fertility, early age of first intercourse, intergenerational sex, and low likelihood of partner circumcision put many young women at increased risk [5]. It is therefore critical to prioritize access to HIV testing, prevention, and treatment for this vulnerable population.

HIV testing services (HTS) and knowledge of HIV status are key steps toward accessing HIV care, treatment, and prevention services. HIV self-testing (HIVST) is a promising approach to increase HTS access and overcomes many of the barriers to HIV testing, especially among young people. With self-testing, an individual collects his or her own sample (oral fluid or blood), performs the test, and interprets the result. A major development in the rapidly growing field of HIVST occurred in December 2016 when the World Health Organization (WHO) recommended HIVST as an additional approach to HTS [6]. For individuals living with HIV, HTS is a critical entry point to life-sustaining care. Successful HIV treatment through antiretroviral therapy (ART) is also essential for prevention of secondary transmission [7]. For those who are HIV negative, HTS provides information about risk reduction strategies and the importance of maintaining HIV negative status. In the 2012 Kenya AIDS Indicator Survey, among those who had not been tested for HIV, 36.7% of women in the age range of 15 to 64 years felt they did not need to be tested, and 17.5% of women were never offered an HIV test [5]. The focus area for the study, Homa Bay County, Nyanza region, is the epicenter for HIV in Kenya, with prevalence at 26% (vs 5.9% for Kenya overall) [8]. Additionally, HIV prevalence in this area is higher for women than men (27.8% vs 24%) [8]. In Nyanza region, 21% of young women and 27% of young men have had sex before the age of 15 years. By 18 years of age, this is two-thirds of both young women and men [9]. From these data, it is evident that there exists a crucial window between 15 and 18 years of age during which young people should access HTS. Highlighting the importance of HTS, WHO recommends it as an important HIV prevention priority, crucial to HIV programmatic success globally [10].

In this GIRLS Study protocol, we describe our planned approaches to address the HIV prevention and treatment continuum that will inform best practices to increase young women’s uptake of HIV prevention, testing, and linkage to HIV treatment services in a high HIV-burden African setting. As this study will be conducted within the framework of implementation science, data generated will help determine which study elements can be scaled up to a national level and will directly inform and support Kenya’s HIV epidemic control targets and approaches [11]. Lessons learned will be shared with stakeholders, including policy makers, implementation scientists, and nongovernmental agencies elsewhere in sub-Saharan Africa who are exploring how best to enhance the HIV prevention and treatment cascade for AGYW.

The study aims are organized by primary and secondary objectives. The primary objectives are to (1) identify the preferred recruitment venue and testing modality that targets and finds the highest number of previously undiagnosed HIV infected and at-risk AGYW in Homa Bay County, Nyanza region, western Kenya (aim 1), and (2) conduct cost-effectiveness analyses to determine the relative utility of the different seek, testing and linkage interventions (aim 3).

The secondary objectives are to (1) pilot and evaluate adaptive interventions to link newly diagnosed HIV positive female youth to treatment and care services (aim 2a), (2) identify barriers and facilitators to seeking HIV care services after receiving a positive diagnosis (aim 2b), (3) identify barriers and facilitators to seeking HIV prevention services for high-risk female youth after receiving a negative test result (aim 2c), and (4) deliver a primary HIV prevention intervention to high-risk HIV negative female youth (aim 2d).

### GIRLS Study Components

We describe in this section the components of the GIRLS Study. Our study is conducted in partnership with the local community in conjunction with an implementing partner, Impact Research and Development Organization (IRDO), which has already successfully delivered combination HIV prevention services to youth in many counties in Nyanza and most recently completed a gender-specific combination HIV prevention study (MP3 Youth; R01AI094607) [12].

#### Mobile Health Delivery of HIV Testing Services

Our implementing partner has utilized health teams in mobile settings for service delivery, and useful community engagement lessons have been learned [12]. Mobile health clinic teams will be composed of a driver, mobilizer, receptionist, laboratory technologist, and research assistant or HTS counselor. Their role will be to do community mobilization and refer potential participants to the mobile event; obtain consent, assess eligibility, perform HTS, and administer computer-assisted personal interviewing (CAPI); draw, process, and ship to external laboratory samples of those who test positive; and refer participants for HIV care and treatment or prevention services, as appropriate. We expect that our study will also ascertain whether mobile health clinic teams or home-based recruitment strategies are preferred by AGYW and which strategy identifies the highest number (yield) of HIV-positive female youth.

#### Home-Based Delivery of HIV Testing Services

Home-based HTS has been provided in Kenya to individuals and families since 2006 and was piloted in Nyanza Province, with a high general acceptance rate above 90% [13]. The home-based and community-based recruitment strategies used in the study is a departure from the more passive approach to HTS that is conducted in antenatal clinics, health facilities, and research clinic settings. HTS and recruitment for research has traditionally been done in health facilities. Due to financial, health system, cultural, and knowledge barriers experienced by adolescents, young women may be less likely to access standard facility-based HTS. Therefore, our study offers HIV testing and recruitment in different settings to inform best practices regarding recruitment and testing strategies targeting this population.

#### HIV Self-Testing

Youth are least likely to get HIV testing, and innovative strategies are needed to reach young women. HIV self-testing has the potential to overcome many of the barriers to HIV testing to increase HTS access. With self-testing, individuals collect their own sample, perform a rapid HIV antibody test, and interpret their results in the absence of a provider. It offers increased convenience, privacy, and autonomy and can normalize regular testing. Existing research shows a high level of acceptability and demand for HIVST across a wide range of populations and settings, as well as good accuracy in the hands of lay users [14-19]. A study conducted in Malawi found that uptake of self-testing was high, especially among adolescents, notably among young women in the age range of 16 to 19 years [20]. However, the acceptability of self-testing and use of this testing strategy among AGYW have not been well documented in other parts of sub-Saharan Africa. Therefore, this study provides an opportunity to expand the knowledge base of how HIVST may contribute as an additional option for youth to know their status. Offering HIVST as a testing option in this study will help assess acceptability and uptake of HIVST among AGYW. In light of WHO’s recommendation for scaling up HIVST and call for additional evidence on ways to operationalize this technology [6], the Government of Kenya’s inclusion of HIVST in their 2015 HTS guidelines [21] and national rollout is timely [22]. The lessons learned from this component of the study will inform Kenya’s Ministry of Health about some of the challenges of making self-test kits available for the general public, as well as for AGYW.

#### Financial Incentives for Uptake of HIV Prevention and Treatment Behaviors

The use of financial incentives to encourage desired behaviors has been explored in sub-Saharan Africa. Interventions such as conditional cash transfer that offer cash or goods to individuals or households on the condition that they meet prespecified conditions such as send their children to school or seek certain health services have shown promise [23-25]. However, unconditional cash transfers (UCTs), which are not tied to any prespecified behaviors have also been found to be effective as an HIV prevention strategy [26,27]. In our study, we offer a one-time UCT for a subset of participants (ie, those who do not link to care after the first randomization in the linkage to care sequential multiple assignment randomized trial [SMART] pilot) because adolescents who test positive may experience structural barriers (eg, transportation costs) in linking to care in a timely manner. Linkage is a one-time event, and we assume that newly diagnosed HIV-positive female youth will link to care within 2 weeks after the first randomization (standard referral vs standard referral and short message service [SMS]). The 2-week time frame for linkage to care is defined by the national government; those identified as HIV-infected should be started on treatment during this period. In the study, we will track whether the participants have linked during this 2-week time frame and will analyze time to linkage to HIV care and treatment services.

#### SMS for Engagement and Retention in Care

Offering linkage to care after a positive HIV test is critical for risk reduction and lifesaving ART. Of Kenyans in the age range of 15 to 65 years self-reported as positive, 79.4% enrolled in care services within 90 days of diagnosis [28]. Previous studies in Uganda and South Africa showed 90% uptake of HTS and linkage among adults, but adolescents were not included; effective strategies need to be identified for adolescents, in particular females, as barriers likely differ. Evidence of SMS effectiveness for supporting linkage to and retention in HIV care exists for adults [29,30]; we will incorporate SMS elements in our study to assess impact for female youth.

Kenya is exploring a new electronic tracking system using SMS text messaging to facilitate linkage to and retention in care and adherence to antiretrovirals for people living with HIV (PLH), as well as to relay prevention messages for high-risk HIV negatives. This national platform envisions use of mobile phones via the SMS platform for communication among facilities, health care workers, and patients to track and support linkage, adherence to treatment, retention, and prevention interventions for improved outcomes. Data generated by this research in the use of an SMS to engage participants (while maintaining their privacy and confidentiality) will inform this planned national platform and guide improvements of the system.

Safeguards for protecting confidentiality of questionnaires and other data will be strictly enforced. We will ensure confidentiality with mobile phone usage by confirming user identity through using an agreed upon code to ensure that the phone user is the study participant before sending an SMS or unstructured supplementary service data messages, as well as airtime reimbursement and digital cash transfers.

## Methods

**Figure 1:**
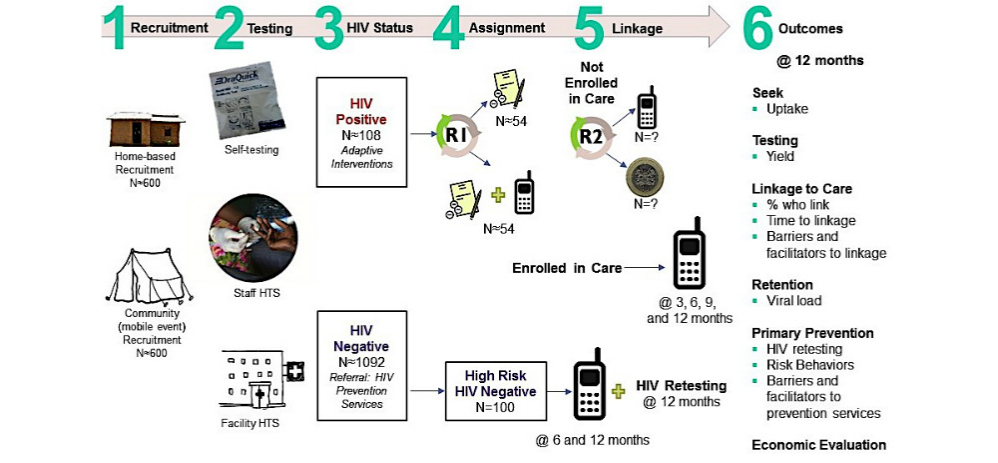
Study design.

The GIRLS Study will evaluate the prevention-treatment continuum interventions to increase uptake of HIV testing, linkage to and retention in care, and prevention among AGYW (see Figure 1). We will compare two *seek* recruitment strategies; three *test* strategies, including a self-testing option; and pilot adaptive *linkage* to care interventions among at-risk AGYW in the age range of 15 to 24 years in Homa Bay County, western Kenya. For participants newly diagnosed with HIV, we will pilot linkage to care interventions to know the best way to support AGYW so that they receive the care and treatment they need to stay healthy. Additionally, we will evaluate a primary prevention messaging intervention to support identified HIV-negative AGYW in reducing HIV risk and adhering to recommended HIV retesting frequency. Exit interviews offered to participants in the HIV-positive and -negative cohorts will explore barriers and facilitators to seeking HIV care services or HIV prevention services, respectively.

### Participants

Participants will be recruited from Homa Bay County, Nyanza region, western Kenya from three subcounties: Homa Bay, Mbita, and Ndhiwa. Recruitment began in the last week of May 2017 and is planned to reach 1200 female youth over an accrual period of up to 12 months, followed by 1 year of follow-up. Eligibility criteria include (1) female in the age range of 15 to 24 years, (2) able to understand spoken English or Kiswahili or Dholuo, (3) resides in Homa Bay County, (4) not previously diagnosed as HIV positive, and (5) willing to provide their informed consent if aged ≥18 years or if in the age range of 15 to 17 years and not an emancipated minor, has a parent or guardian willing to provide consent, in addition to the minor’s assent.

All study methodology has been approved by both the Kenyatta National Hospital/University of Nairobi Ethics and Research Committee (#P491/07/2015) and Yale University’s Human Investigation Committee (#1512016985).

To improve the health and well-being of AGYW, it is vital to understand which testing services female youth prefer and whether they prefer receiving services at home, in the community outside the health facilities, or in the health facilities. This study focuses on aims 1 and 2; study methodology is described in further detail below.

### Recruitment Strategies (Aim 1)

**Figure 2:**
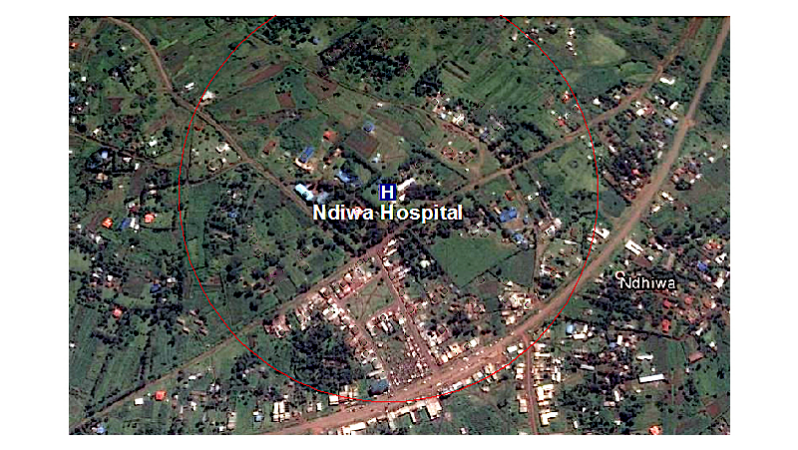
Sampling method.

Home-based and community-based recruitment strategies will be utilized to determine which strategy identifies the highest number of previously undiagnosed HIV infected and at-risk AGYW. In the home-based strategy, research assistants provide information about the GIRLS Study and enroll participants in their homes. In the community-based strategy, temporary structures such as tents are set up, and participants are enrolled during mobile health clinic events. We will stratify the sample by the three study subcounties: Homa Bay, Mbita, and Ndhiwa. Mapping of catchment areas surrounding health facilities in the three subcounties was used to establish relationships with referral clinics. We will select a random starting point that is within walking distance (about 5 km) of the catchment area of the referral health facility (see Figure 2). In each stratum, the number of participants to be recruited will be proportional to the number of AGYW in the age range of 15 to 24 years living in the respective study catchment area. Participants will be recruited through two recruitment approaches (home-based or mobile health clinic event). Both recruitment strategies will run concurrently until the desired sample size is reached. If needed for sample size attainment, we will expand the sampling area to a 6 to 10 km circumference of the referral clinic.

### HIV Testing Strategies (Aim 1)

Whether recruited in the home or at the mobile health clinic event, all participants will be offered three HIV testing options: (1) oral fluid-based HIV self-testing, (2) staff-aided testing per Government of Kenya testing guidelines, or (3) referral for standard facility-based HTS (see Figure 1). After completing the testing modality of their choice, participants will receive an SMS survey within 2 weeks to learn about their testing experience. After completing the survey, all participants will receive airtime reimbursement of KSh 100 (approximately US $1). For those who choose self-testing, participant will take a photo of the test result on a study phone (provided by the study), which will be collected by a study staff after the participant informs the study staff of test completion. During a follow-up visit, the study staff will also perform confirmatory testing for all participants with a positive or indeterminate result, as well as a proportion of participants with a negative self-testing result. The study staff will also collect the used self-test kit and conduct a brief interview on participant experience with self-testing and interpretation of results. Participants who choose referral to the health facility will be tested by the facility staff and testing completion confirmed by study staff from health records. In this way, the primary outcome of an HIV test conduction and result will be achieved as part of the *test* element of the study.

### Adaptive Linkage to Care Pilot for AGYW Living With HIV (Aim 2)

**Figure 3:**
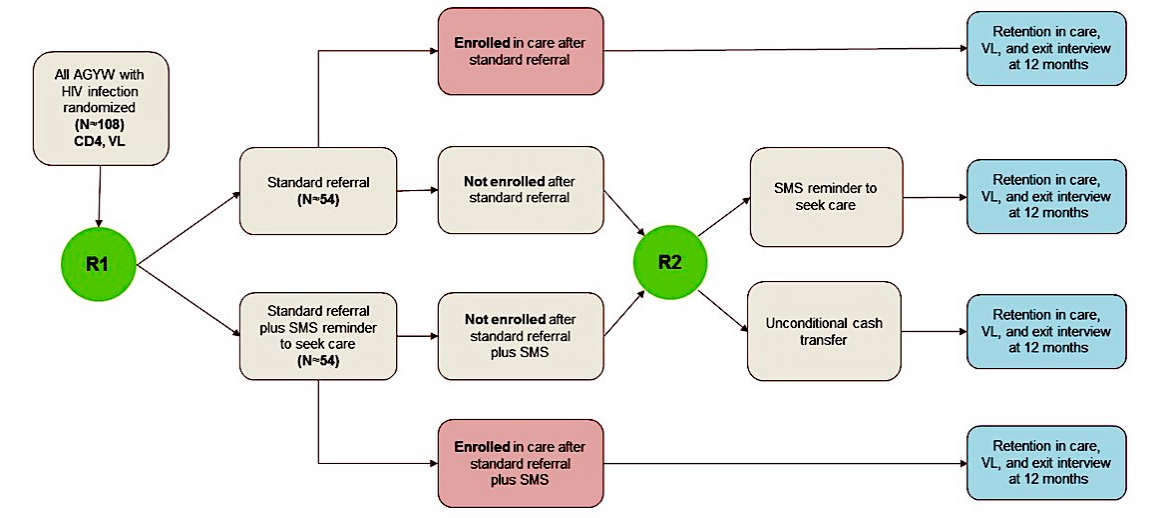
Sequential multiple assignment randomized trial (SMART) design to compare adaptive interventions.

All participants who are HIV positive per the Government of Kenya testing guidelines [31] will be invited to enroll in the SMART pilot to compare adaptive linkage to care interventions. Viral loads will be collected before enrollment in the linkage pilot. Additional criterion for participation in the cohort is a willingness to receive SMS texts to link to HIV care and treatment services. Participants will be given a study phone to ensure that lack of phone access is not a barrier to enrollment in the cohort. Participants enrolled in the pilot will undergo sequential multiple assignment randomization (SMART design; see Figure 3), with provision of the subsequent intervention based on participant response to the initial intervention [32]. The initial randomization is to standard referral (counseling and a referral note) or standard referral plus SMS. Some participants may seek HIV care after receiving the standard referral or standard referral plus SMS. Those who do not link to care within 2 weeks after the initial intervention will then be randomized to receive either an SMS text message with linkage encouragement or a one-time financial incentive equivalent to average transport cost to the health facility.

### Randomization

We will utilize an approach called permuted blocks randomization for participant assignment to the SMART trial linkage to care intervention. Permuted block randomization has several advantages, one of which is promoting equal distribution of participants in each group. Block randomization is designed to ensure sample size balance across study conditions over time and supports allocation concealment. However, block randomization does not attempt to adaptively balance study conditions on prognostic covariates. A list of random allocations will be generated in randomly ordered permuted blocks of either 2, 4, 6, 8, or 12 allocations. The allocation list will be stored in a password-protected file that will only be accessible to the study statistician, data manager, and project manager.

The field study coordinator will provide a list of study identification numbers of participants needing allocation. The study statistician or data manager will record the unique study identification number and the date of the assignment on the allocation list and relay the assignment to the field study coordinator. Alternatively, if we find that this is not efficient during implementation of our study, we will pursue other methods such as using sequentially numbered, sealed envelopes containing participant allocation that may be prepared beforehand [33].

We anticipate approximately 108 AGYW living with HIV to be identified and randomly assigned to either standard referral or standard referral plus SMS at the first randomization. Participants not linked to care after the first randomization will be rerandomized to receive either an SMS text message or financial incentive.

### AGYW Successfully Linked to Care

Participants who successfully link to care, defined as date of first appointment at a comprehensive care clinic, will receive motivational SMSs with adherence to medication and care messages and an SMS survey at intervals of 3 months for 12 months. Participants will receive KSh 100 (approximately US $1) every time they complete the SMS surveys. At 12 months, we will follow up with those participants who tested positive and enrolled in care to measure HIV viral load to determine whether they were linked, retained in care, and adherent to treatment. We will also conduct an interview at 12 months to determine barriers and facilitators for linkage to HIV care and treatment. At this final study visit, participants will receive KSh 400 (approximately US $4) as reimbursement for their time and transportation costs.

### Prevention Intervention for HIV Negative Cohort (Aim 2)

Beyond identifying PLH and linking them to care, primary HIV prevention efforts are also essential. Our study will evaluate a primary prevention messaging intervention to support identified high-risk HIV-negative young women in reducing HIV risk and adhering to HIV retesting recommendations. A *high risk* determination is made if the participant is sexually active and meets one or more of the following: (1) sexual partner is HIV positive or of unknown HIV status, (2) reports 3 or more sexual partners in the last month, or (3) reports that her sexual partner has multiple sexual partners. Participants will be given study phones to ensure that lack of phone access is not a barrier to enrollment in the cohort. The messaging intervention will be a health promotion message. A follow-up survey will collect information on HIV risk behaviors, condom use, and assess HIV retesting behaviors and intentions. Participants enrolled in the HIV-negative cohort will receive airtime reimbursement of KSh 100 (approximately US $1) every time they complete SMS surveys (at 6 and 12 months). At 12 months, participants in the prevention intervention will be retested for HIV and will also complete a face-to-face interview on barriers and facilitators to accessing HIV prevention services. They will receive KSh 400 (approximately US $4) as reimbursement for their time and transportation costs.

Study intervention descriptions are outlined in Textbox 1. For the logic model, see Multimedia Appendix 1.

### Data Collection

#### Computer-Assisted Personal Interviewing

Cross-sectional sociodemographic, HIV prevention, health services access, and other behavioral data will be collected at baseline from all females using a staff-administered CAPI. The CAPI data will contribute to better understanding of specific behavioral risks, HIV testing, and prevention intervention exposure for female youth enrolled in the study. Participants who select the self-testing option will complete an additional CAPI survey that will capture their experience with self-testing and interpretation of the results. Participants enrolled in the HIV-positive and -negative cohorts will undergo CAPI interviews to identify barriers and facilitators to seeking HIV care and treatment or prevention services, respectively.

**Study intervention descriptions.**

All female youth (N=1200)

- Human immunodeficiency virus testing services (HTS) via home-based or mobile health clinic event–based recruitment strategies
- HIV testing approaches that females can choose from include (1) oral fluid HIV self-testing at their convenience, (2) staff-aided testing at home or mobile site, or (3) a referral to a health care facility where HIV testing will be done by a health care provider (standard facility-based HTS)
- Behavioral survey at baseline visit
- SMS (short message service) HIV test experience satisfaction survey
- Staff-administered questionnaire about self-testing for females who choose self-testing option

HIV-positive cohort (N≈108)

- Point-of-care cluster of differentiation 4 cell count at baseline
- Viral load testing using dried blood spot at 0 and 12 months
- *Referral for care and treatment; Adaptive linkage to care interventions.* All HIV-positive participants (N≈108) will be randomized to receive standard referral (N≈54) or standard referral plus SMS text message (N≈54) to link to HIV care and treatment services; those who do not link to care within 2 weeks will be rerandomized to an SMS text message or an unconditional cash transfer. We will confirm linkage by self-report via phone call and verified by medical record review
- Antiretroviral therapy adherence and treatment SMS reminders, combined with health status surveys at 3, 6, 9, and 12 months aligned to school holidays for those in school, when the participants would have access to phones
- Face-to-face interview on barriers and facilitators to seeking HIV care services after receiving a positive diagnosis at 12 months

HIV-negative cohort (N=100)

All HIV-negative females will receive the following: risk assessment counseling, condoms, and referral for other prevention services (partner and family testing, pre-exposure prophylaxis, other family planning (contraception) methods, sexually transmitted infection screening and treatment, drug use counseling and other mental health services, social and nutritional support, legal services, sexual and gender based violence services, and others that might be identified during the interview or risk assessment with participants

A subset of randomly selected high-risk (risk score ≥2 as defined by eligibility criteria for high risk) HIV-negative females (N=100) will receive:

- An SMS text message at 6 and 12 months with a health promotion message
- SMS survey to collect HIV risk behaviors, condom use, and willingness to retest for HIV at 6 and 12 months
- HIV test at 12 months
- Face-to-face interview on barriers and facilitators to accessing HIV prevention services at 12 months

#### SMS Surveys

The SMS surveys will capture the following information: (1) HIV testing option and test experience, (2) successful linkage, (3) sexual risk behaviors, (4) retention in care, (5) adherence barriers and facilitators, (6) willingness to retest (for subset of HIV negatives), and (7) status disclosure.

### Power Analysis

Power calculations are based on the yield of newly diagnosed HIV infected across recruitment strategies and testing options offered to female youth (aim 1). Therefore, we did not approach this power analysis from the perspective of calculating a sample size needed to detect a particular effect size with at least 80% power. Rather, because this part of the study (aim 1) is not a clinical trial but an exploration of preferences and yield of recruitment and participant-selected testing strategies, we started with the large, feasible sample size and determined the size of effects that could be detected with at least 80% power when comparing recruitment strategies and HIV testing approaches using version 12 of the Power Analysis and Sample Size Software program [34] for logistic regression.

The expected percentage of AGYW testing positive is based on our MP3 Youth pilot study conducted in the same region [12]. In that study, 55 of 636 female youth not previously diagnosed were found to have HIV infection (data not yet published). Thus, we expect about 9% of all tested females in this study to have HIV infection not previously diagnosed. With this estimate of the expected percentage of girls with newly diagnosed HIV infection, we asked how small of an increase in the odds of newly diagnosed HIV infection could be detected with 80% power when community-based recruitment is compared with home-based recruitment. If approximately 600 youth are recruited by each strategy, power is 80% to detect an odds ratio (OR) of 1.67; in other words, a modest increase from 9% to 14% newly diagnosed with HIV infection. When comparing testing approaches and assuming an equal proportion of girls prefers each approach and testing approach does not interact with recruitment strategy, power is 80% to detect an OR of 1.85. If there is an interaction between recruitment strategy and testing approach when comparing testing approaches within a recruitment strategy (ie, estimating simple main effects), power is 80% to detect an OR of 2.32.

These calculations show that our sample size (N=1200) can detect modest differences in the yield of newly diagnosed HIV infection across both recruitment strategies and three testing approaches. Among the approximately 108 female youth we expect to be newly diagnosed with HIV infection, about 20.4% (22/108) are expected to successfully initiate HIV care at a health facility after standard referral plus a single SMS text message. The remaining 79.6% (86/108) who are not linked to care after the initial SMS text message will be randomly assigned to either SMS text messages or to an UCT. Assuming at least 60% (52/86) are linked to care at some point during follow-up, survival analysis employing Cox regression will be able to detect a hazard ratio of 2.2, describing the impact of treatment condition (SMS vs UCT) on the occurrence and timing of successful linkage to care at a health facility with 80% power.

### Outcome Measures

For the analysis plan, see Multimedia Appendix 2.

The key outcomes we detail below are broken out by study aims and based on the HIV prevention and treatment continuum that organizes our outcomes of interest by recruitment strategies, testing, linkage, retention, and primary prevention.

**Figure 4:**
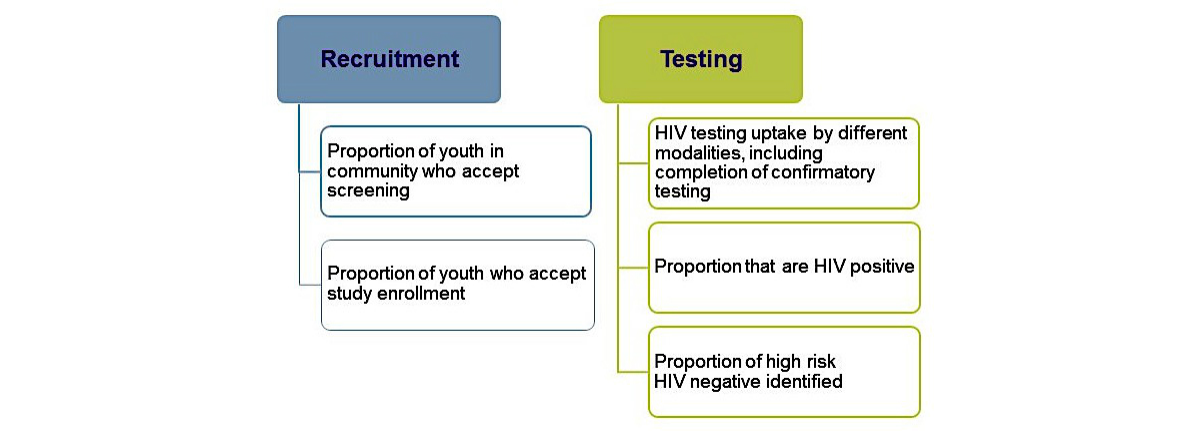
Aim 1 outcome measures.

The primary end point for comparisons of recruitment and testing strategies (aim 1) is newly diagnosed HIV infection. Additional outcomes of interest for aim 1 are outlined in Figure 4.

We will determine which HIV testing approaches are most preferred among all female youth and among high-risk female youth. We will also determine which recruitment and HIV testing approaches yield the highest rates of newly diagnosed HIV infection. Multinomial logistic regression will be used to understand which testing approaches are most preferred or used by female youth. Preference for HIV testing at a health facility will be the reference category for the dependent variable. A model with only an intercept will be used to estimate the proportion of girls preferring each testing approach and to test for pairwise differences in preferences (eg, does a higher proportion of girls prefer staff-aided rather than self-testing?). In addition to overall preferences, we will consider age, risk behaviors, and HIV testing history as predictors of preferences by adding participant characteristics to the multinomial logistic regression analysis. Type of testing selected by participants, including refusal to test, will be regressed on predictors to estimate how characteristics of female youth impact HIV testing preferences. Model coefficients will describe the change in odds of preferring other types of testing relative to the reference category (facility-based testing).

Logistic regression analysis will be used to determine which recruitment and HIV testing approaches yield the highest proportion of newly diagnosed HIV infection. HIV testing result (negative vs positive) will be regressed on variables indicating recruitment strategy (home vs mobile health clinic event) and type of HIV testing (oral self-testing, staff-aided, or facility-based). With facility-based testing following community-based (mobile event) recruitment as a reference category, we will estimate how home-based recruitment and other testing approaches change the odds of a positive test result. The interaction between recruitment strategy and HIV testing approach will be tested to determine whether the yield of each testing approach is conditional on recruitment strategy. If a significant interaction effect is found, simple main effects of testing approach within each recruitment strategy and simple main effects of recruitment strategy within each testing approach will be estimated. In addition to comparing yield by recruitment strategies and testing approaches, we will consider interaction effects with youth characteristics such as age, risk behaviors, and HIV testing history and type of testing. Similar logistic regression analyses will examine differences in the odds of completing all confirmatory testing by recruitment strategy and approach to testing.

Home-based and community-based recruitment strategies will also be compared to determine which strategy is more effective in reaching higher risk female youth. Rates of acceptance of screening among potential participants offered screening and rates of enrollment among eligible participants will be compared by recruitment strategy using logistic regression. Risk will be characterized by number and serostatus of sex partners, concurrency, condom use, and patterns of HIV testing by the participant and any sex partners.

**Figure 5:**
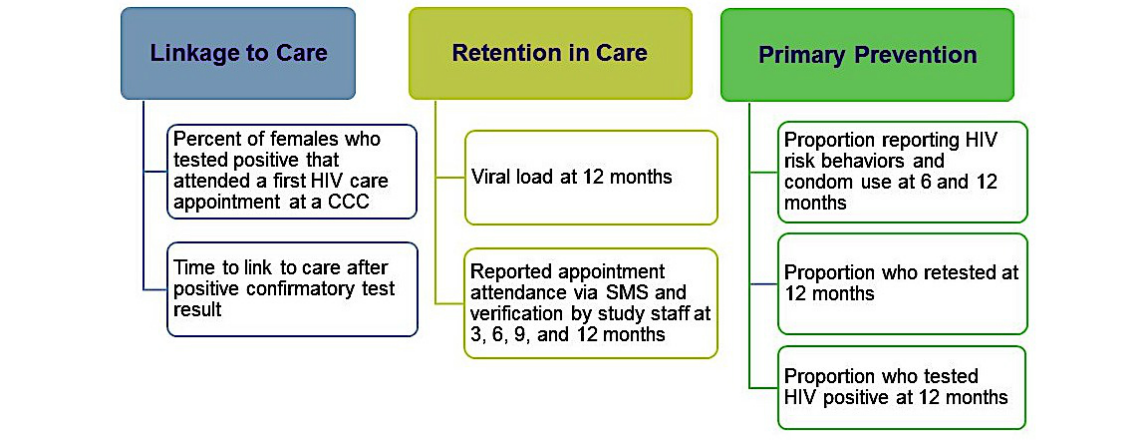
Aim 2 outcome measures.

Outcomes of interest for aim 2 are outlined in Figure 5.

Descriptive statistics and survival plots will be used to characterize occurrence and timing of linkage among AGYW responding to the initial SMS text message and AGYW in each augmented treatment condition. Survival analysis [35,36] will determine which approach to augmenting linkage—SMS or economic incentive—is more effective in linking female youth newly diagnosed with HIV to care sooner. Using the Cox proportional hazards model, difference in the likelihood and timing of linkage between treatment conditions will be quantified with an interval estimate of the hazard ratio. Median time to linkage also will be estimated and compared across treatment conditions.

**Figure 6:**
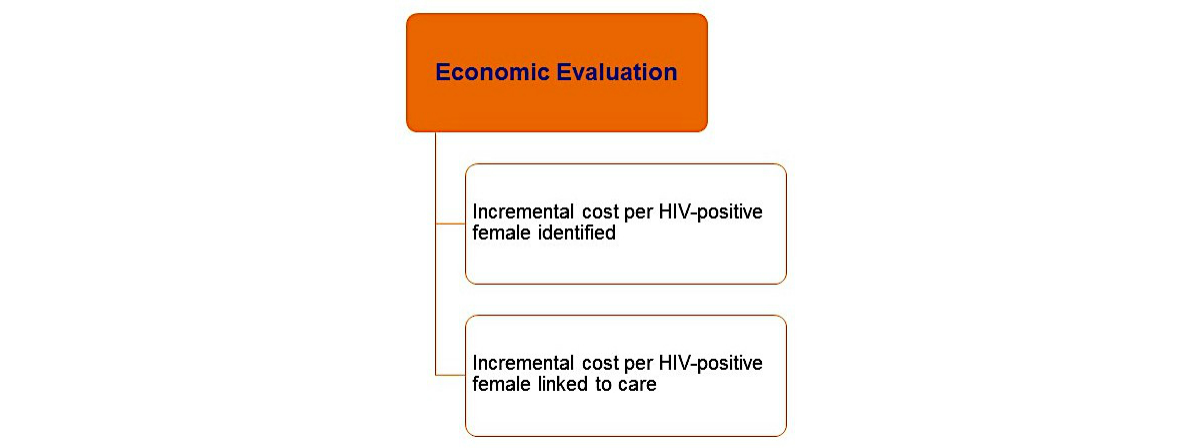
Aim 3 outcome measures.

For the prevention intervention, we will assess risk behaviors, condom use, additional HIV testing, linkage to prevention, and any new HIV diagnoses over the year since study enrollment. Retesting, prevention service use, and new HIV will be examined in relation to recruitment strategy, testing approach at enrollment, and participant risk behavior and other characteristics. Key outcomes of interest for aim 3 economic evaluation to determine the comparative cost effectiveness of the seek, testing, and linkage interventions are outlined in Figure 6.

To determine costs, we will conduct a microcosting of the cost per HIV-infected person identified under each *seek* and *test* strategy. This will be followed by an incremental cost-effectiveness analysis of the linkage to care interventions among the HIV-infected and a cost analysis of the SMS intervention among HIV-uninfected AGYW. These costs will include all costs required to deliver each intervention (eg, home visits, staff time, supplies, and monitoring). Data will primarily utilize a microcosting methodology that uses project expenditure and management records, employing structured costing spreadsheets that record each resource, category (eg, personnel and supplies), quantity (eg, hours), and unit costs. The cost data for the self-test group will include costs of obtaining a confirmatory test for those with positive self-testing results. The cost data will be stratified by HIV testing venue (home-based, community-based (mobile), or referral facility). With these cost data, we will calculate and compare the cost per HIV-infected person identified in each HIV testing strategy. We will also evaluate the costs of the linkage interventions per individual successfully linked to care. The results of the economic evaluation will allow us to identify the most efficient, affordable way to seek high-risk young women and test them. For the linkage interventions, we will utilize the cost data as well as the effectiveness results to compute the incremental cost-effectiveness ratio (ICER) for the novel linkage intervention that is used.

The ICER will reveal whether the costs of achieving the incremental gains from additional linkage interventions suggest whether it is cost-effective or not. These results will provide novel data on the cost-effectiveness of linkage to care interventions for high-risk females in sub-Saharan Africa.

## Results

We are currently in the recruitment phase of our study and anticipate accrual to be completed by summer 2018, and final study visits to be completed by 2019. We expect to enroll 1200 participants overall, with a random selection of 100 high-risk HIV-negative AGYW for the SMS prevention intervention (HIV-negative cohort) and approximately 108 AGYW who are living with HIV for the SMART design pilot of adaptive linkage to care interventions (HIV-positive cohort). Findings will determine the preferred recruitment strategy and testing option that finds the highest number of previously undiagnosed AGYW living with HIV and at-risk female youth. We anticipate that the linkage to care interventions for AGYW living with HIV and the prevention intervention for high-risk female youth will be feasible and acceptable to implement.

## Discussion

To reach the 90-90-90 targets set by the Joint United Nations Programme on HIV and AIDS, innovative strategies are needed to reach adolescent girls and young women, a priority population at significant risk for HIV infection. Increasing knowledge of HIV status is the entryway into care and prevention services. Despite the need to increase knowledge of HIV status in this priority population, access, coverage, and uptake remain poor [1-3]. Data show that we are getting closer to, but still not reaching, the most vulnerable populations at a coverage level that will curb the HIV epidemic. Our findings will inform best practices to increase young women’s uptake of HIV prevention, testing, and linkage to HIV treatment services in a high-HIV-burden African setting. As this study will be conducted within the framework of implementation science, data generated will help determine which study elements can be scaled up to a national level and will directly inform and support Kenya’s HIV epidemic control. Study components include using mobile health clinic events (outreach) and home-based delivery of HTS, self-testing as an innovative strategy for expanding testing among youth, UCTs, and utilizing an SMS for engagement and retention in care. This study will provide additional insights to push programming closer to achieving 90-90-90 for adolescent girls and young women.

A study limitation is the small sample size of the SMART design pilot to compare adaptive interventions for AGYW living with HIV. Strengths of the study include the longitudinal cohorts (ie, HIV-positive and -negative cohorts), which will allow estimation of potential intervention impact over time in this important population.

## Appendix

Multimedia Appendix 1 Logic model.

Multimedia Appendix 2 Evaluation plan.

## Abbreviations

AGYW: adolescent girls and young women
ART: antiretroviral therapy
CAPI: computer-assisted personal interviewing
HIVST: HIV self-testing
HTS: HIV testing services
ICER: incremental cost effectiveness ratio
IRDO: Impact Research and Development Organization
OR: odds ratio
PLH: people living with HIV
SMART: Sequential Multiple Assignment Randomized Trial
SMS: short message service
UCT: unconditional cash transfer
WHO: World Health Organization
