# Supplementary material for: High-Yield HIV Testing, Facilitated Linkage to Care, and Prevention for Female Youth in Kenya (GIRLS Study): Implementation Science Protocol for a Priority Population
Source: JMIR Res Protoc. 2017 Dec 13;6(12):e179. doi: 10.2196/resprot.8200 (PMC5754209; doi:10.2196/resprot.8200)
Supplement: Multimedia Appendix 1 [file resprot_v6i12e179_app1.pdf]

Appendix 1. Logic Model

<table>
<tr><th>Study Objectives</th><th>Resources and Inputs
(what we invest)
In order to accomplish our activities, we will need the following:</th><th>Activities
(what we do)
In order to address HIV prevention, we will conduct the following activities:</th><th>Outcomes for Aims 1 & 2
We expect that once completed or under way, these activities will produce the following evidence along the HIV prevention and treatment continua:</th><th>Outcomes for Aim 3</th><th>Long-term Outcomes and Impact
We expect that once completed our study will have the following impact</th></tr>
<tr><td rowspan="3">Aims 1 and 2 Objectives:

(1) To determine the preferred recruitment venue and testing modality that targets and finds the highest number of HIV-infected and at-risk female youth in the age range of 15 to 24 years in Homa Bay County, Nyanza region, western Kenya. This will be determined by examining uptake and yield of previously undiagnosed HIV infection of the two “seek” strategies (community or home-based) and the three “test” strategies (self-testing, HTS in a home or mobile setting, or facility-based HTS) among female youth;

(2) To pilot and evaluate adaptive interventions to link newly diagnosed HIV positive female youth to treatment and care services; and

(3) To identify barriers and facilitators to seeking HIV care services after receiving a positive diagnosis.</td><td>Funding</td><td>Human Subjects Approval</td><td>Aims 1 and 2 Outcomes:</td><td>Aim 3 Outcomes:</td><td>Increased HIV testing and counseling</td></tr>
<tr><td>Community engagement</td><td>Hire and train field staff</td><td><u>Recruitment:</u>
Ability to identify and recruit female youth using two seek strategies (home-based vs. community-based)
-The proportion of youth in the community who accept study screening (estimated from total youth approached as the denominator);
-The proportion of youth who accept study enrollment (estimated from youth screened and eligible as the denominator);</td><td>Conduct comprehensive economic evaluation to determine the comparative cost effectiveness of the seek, testing and linkage interventions.</td><td>Increased correct and consistent condom use</td></tr>
<tr><td>Scientific team</td><td>Enroll participants.</td><td><u>Testing</u>:
-Testing uptake by different testing modalities;
-Completion of confirmatory testing;
-Proportion that are positive;
-Among those who are positive, completion of baseline collection of CD4 and viral load tests;
-Proportion that are high-risk negative.</td><td><u>Seek</u>: Compute the cost per HIV-infected female youth identified under each “seek” strategy (home-based vs. community-based)</td><td>Increased treatment, ART adherence, and reduced HIV</td></tr>
</table>

(4) To deliver an HIV prevention intervention to a randomly selected subset of high risk negatives.<br><br>(5) To identify barriers and facilitators to seeking HIV prevention services for high risk female youth after receiving an HIV negative test result.					
**Aim 3 Objectives:**<br><br>To conduct an economic evaluation to determine the comparative cost effectiveness of the seek, testing and linkage interventions.	Established local implementing partner	Pilot adaptive linkage interventions.	**Linkage to Care:**<br>-Percent of females who tested positive that attended a first HIV care appointment at an HIV Comprehensive Care Center<br>-Time to link to care after positive confirmatory test result.	**Test**: Compute the cost per HIV-infected female youth identified under each "test" strategy (self-testing, staff delivered HTS testing, standard facility-based HTS);	Increased use and familiarity with oral-fluid HIV self-testing kits.
	Protocol	Follow up cohorts	**Retention in Care:**<br>-Completion of viral load testing at 12 months;<br>-Reported appointment attendance via SMS and verification by study staff at 3, 6, 9, and 12 months.	**Link**: Calculate the incremental cost-effectiveness per HIV positive female linked to care;	
	Training	Disseminate results	**Primary Prevention:**<br>-Proportion reporting HIV risk behaviors, and condom use at 6 and 12 months;<br>-Proportion who re-tested at 12 months;<br>-Proportion who tested positive at 12 months.	**Prevention**: Compute the cost of the SMS prevention intervention among HIV negative female youth.	
	Local clinics				
