# Supplementary material for: High-Yield HIV Testing, Facilitated Linkage to Care, and Prevention for Female Youth in Kenya (GIRLS Study): Implementation Science Protocol for a Priority Population
Source: JMIR Res Protoc. 2017 Dec 13;6(12):e179. doi: 10.2196/resprot.8200 (PMC5754209; doi:10.2196/resprot.8200)
Supplement: Multimedia Appendix 2 [file resprot_v6i12e179_app2.pdf]

Appendix 2. Evaluation Plan

**Aims 1 and 2: Outcomes evaluated based on HIV prevention and treatment continua**	**Measures (Sources)**	**Data Collection Timeline**	**Data Analysis**	**N**
**Recruitment**: Ability to recruit female youth using two seek strategies (home-based vs. community-based). The proportion of youth in the community who accept study screening; the proportion of youth who accept enrollment.	CAPI baseline; (youth participants, total approached)	Baseline	Frequencies; differences in proportion by age, and recruitment strategy; which recruitment strategy yielded the highest rates of newly diagnosed HIV infection. Rates of acceptance of screening among potential participants offered screening and rates of enrollment among eligible participants will be compared by recruitment strategy using logistic regression. Risk will be characterized by number and serostatus of sex partners, concurrency, condom use, and patterns of HIV testing by the participant and any sex partners.	N=1200
**Testing**: HIV testing uptake; completion of confirmatory testing; proportion who are HIV positive; proportion of high risk HIV negative identified.	GIRLS study project database	Baseline, 2 week SMS test experience satisfaction survey.	Frequencies; multiple logistic regression for which testing approach most preferred/used by females. Age, risk behaviors, HIV testing history as predictors of preferences; which testing approach yielded the highest rates of newly diagnosed HIV infection.	N=1200
**Linkage to Care**: Percent of females who tested positive that attended a first HIV care appointment at a CCC; time to link to care after positive confirmatory result.	GIRLS study project database, medical record review at CCC, exit interviews.	2 weeks after first randomized intervention; 1 week to re-randomize; 2 weeks after second randomization (~5 weeks total from start of linkage pilot). Exit interview collected at 12 months.	Descriptive statistics; survival plots for occurrence and timing of linkage. Cox proportional hazards model for differences in likelihood and timing of linkage between the two treatment conditions; median time to linkage. Predictors of successful linkage to care.	N≈108
**Retention in Care**: Completion of viral load testing one year after positive confirmatory test; self-report of appointment attendance. Review of medical records to confirm self-report of clinic attendance.	SMS surveys, medical record review, exit interviews.	3, 6, 9, and 12 months for SMS surveys. Medical record review to confirm clinic attendance self-report. VL and exit interview collected at 12 months.	Frequencies; assess number retained in care at 12 months. Proportion who complete viral load testing at 12 months.	N≈108
**Primary Prevention**: HIV risk behaviors, condom use, and HIV retesting of a sub-sample of high risk HIV negatives	SMS surveys<br>GIRLS study project database, exit interviews.	6 and 12 months for SMS surveys. At 12 months, exit interview and retesting	Descriptive statistics; new HIV diagnosis at 12 months.	N=100
**Aim 3: Economic evaluation**	**Measures (Sources)**	**Data Collection Timeline**	**Data Analysis**	
**Economic evaluation** to determine incremental cost-effectiveness of the seek, testing and linkage interventions	GIRLS study project database	Accrual and follow-up period.	Micro-costing of the cost per HIV infected person under each seek and test strategy. Cost-effectiveness outputs are incremental cost per HIV positive person identified, and incremental cost per HIV positive person linked to care.	
